# Interleukin-35 Suppresses Interleukin-9-Secreting CD4^+^ T Cell Activity in Patients With Hepatitis B-Related Hepatocellular Carcinoma

**DOI:** 10.3389/fimmu.2021.645835

**Published:** 2021-06-10

**Authors:** Qian Zhang, Lanlan Yang, Siqi Liu, Mengyao Zhang, Zhenjing Jin

**Affiliations:** Digestive Diseases Center, Department of Hepatopancreatobiliary Medicine, The Second Hospital, Jilin University, Changchun, China

**Keywords:** interleukin-35, interleukin-9, T cells, hepatitis B virus, hepatocellular carcinoma

## Abstract

Chronic hepatitis B virus (HBV) infection induces dysfunction of immune response and chronic liver damage. However, the mechanisms that account for HBV-related hepatocellular carcinoma (HCC) are poorly understood. The aim of present study was to investigate the modulatory role of interleukin (IL)-35, an immunosuppressive cytokine, to IL-9-secreting T cells in hepatitis B-related HCC. Twenty-two HBV-related HCC patients, twenty-seven chronic hepatitis B (CHB) patients, and eleven controls were enrolled. Serum IL-35 and IL-9 concentration was measured by ELISA. Peripheral and liver-infiltrating non-specific and HBV-specific Th9 and Tc9 cells were assessed by flow cytometry. The regulatory activity of IL-35 to peripheral and liver-infiltrating Th9 cells was assessed in co-culture system between CD8^+^ T cells and HepG2.2.15 cells. Serum IL-35 was up-regulated, while IL-9 was down-regulated in HBV-related HCC patients compared with in CHB patients and controls. Peripheral non-specific and HBV-specific Th9 cells, but not Tc9 cells, were decreased in HBV-related HCC patients. Liver-infiltrating non-specific and HBV-specific Th9 cells were also reduced in HCC tumor sites. CD8^+^ T cells from CHB and HBV-related HCC patients revealed decreased cytotoxicity compared with those from controls. Autologous Th9 cells mediated the elevation of CD8^+^ T cell cytotoxicity, and this process was depending on IL-9 secretion. Recombinant IL-35 stimulation inhibited IL-9 secretion and PU.1 mRNA expression in non-specific and HBV-specific Th9 cells, leading to the suppression of Th9-mediated CD8^+^ T cell cytotoxicity in CHB and HBV-related HCC patients. Our current data indicated that IL-35 might dampen non-specific and HBV-specific Th9 cells activity in HBV-related HCC patients.

## Introduction

Hepatitis B virus (HBV) infection is still a severe public health problem, with approximate 350 million previous and persistent infections all over the world (1). Chronic HBV infection always leads to liver inflammation and fibrosis, resulting in end-stage liver diseases, such as decompensated cirrhosis, liver failure, and hepatocellular carcinoma (HCC) (2). Importantly, hepatitis B is still the most pivotal risk factor for HCC in China, and the outcome of hepatitis B is closely related to the interaction between viral replication and host immune response (3). Acute HBV infection in adults induces multi-specific T cell-responses, which are important for controlling viral infection. In contrast, HBV infection in infants or children always leads to chronic infections, and manifests as immunotolerance or hyporesponsiveness, which was also the characteristic of HCC patients (4). However, the mechanism of immunodysregulation in HBV-induced HCC remains not completely understood.

Naïve CD4^+^ T cells differentiate into different T helper (Th) cells by various transcriptional factors and cytokines induction. Interleukin (IL)-4 and transforming growth factor-β (TGF-β) reprograms the differentiation of Th2 cells, losing Th2 characteristic profile and switching to IL-9 secretion. This process drives a distinct population of IL-9-producing CD4^+^ T cells, which are named as Th9 cells (5). Meanwhile, CD8^+^ T cells also differentiate into distinct subpopulations. IL-9-producing CD8^+^ T cells, which defined as Tc9 cells, are characterized by specific interferon-γ (IFN-γ) and IL-10 expression as well as by low cytotoxicity in response to IL-4 plus TGF-β activation (6). IL-9-producing T cells and IL-9 secretion contributes to tumor immunity and immune-related diseases, which provides useful insight into pathogenesis and treatment (7, 8). Cui et al. revealed that circulating Th9 cells was notably lower in chronic hepatitis B (CHB) patients, and no significant differences in Tc9 percentage between CHB patients and controls (9). However, the profile of viral specific Th9 and Tc9 cells and regulation of IL-9 secretion in HBV-related HCC was not reported.

IL-35 is a newly identified IL-12 cytokine family member, and comprises two heterodimeric subunits, IL-27 β chain Epstein-Barr virus-induced gene 3 (EBI3) and IL-12 α chain p35 (IL-12p35). IL-35 is mainly secreted by regulatory T cells and regulatory B cells, which contribute to immunotolerance and viral persistence during chronic HBV infection (10, 11). Our previous studies revealed that IL-35 regulated CD4^+^ and CD8^+^ T cell function, and played an immunosuppressive role in chronic HBV infection and non-viral hepatitis-related HCC (12–14). A recent study by Zhang et al. showed that IL-35 promoted Th9 cell differentiation in IgG4-related disorders (15). Thus, we hypothesized that IL-35 could also regulate Th9 cells in hepatitis B-related HCC. To test this possibility, we investigated peripheral and tumor-infiltrating Th9 and Tc9 cell population, and assessed the effect of recombinant human IL-35 on non-specific and HBV-specific Th9 cell function *in vitro* in CHB and hepatitis B-related HCC patients.

## Materials and Methods

### Studied Subjects

This study was carried out in accordance with the recommendations of Ethics Committee of The Second Hospital of Jilin University with written informed consent from all subjects. All subjects gave written informed consent in accordance with the Declaration of Helsinki. The protocol was approved by the Ethics Committee of The Second Hospital of Jilin University. Twenty-seven of HLA-A2 restricted CHB patients and twenty-two of HLA-A2 restricted hepatitis B-related HCC were enrolled in the current study. All patients were hospitalized or followed-up in The Second Hospital, Jilin University. Inclusive criteria for CHB patients (1): Positive for HBV DNA and HBV surface antigen (HBsAg) for more than six months. (2) Treatment-naïve to nucleos(t)ide analogue and IFN-α. (3) Alanine aminotransferase (ALT) > 80 IU/L. (4) Alpha fetoprotein (AFP) < 200 ng/mL. (5) Confirmed free from HCC by ultrasound test or computed tomography (CT) scan. Inclusive criteria for hepatitis B-related HCC patients: (1) Positive for HBV DNA and HBsAg for more than six months. (2) Treatment-naïve to nucleos(t)ide analogue and IFN-α. (3) AFP > 400 ng/mL. (4) Confirmed for HCC by contrast enhanced CT or magnetic resonance imaging scan. Exclusive criteria: (1) Co-infected with other hepatovirus. (2) Co-infected human immunodeficiency virus (HIV). (3) Afflicted with autoimmune diseases. (4) Afflicted with other malignance diseases. (5) Pregnancy. (6) Received chemotherapy, radiotherapy, or immunomodulatory therapy before baseline sampling. For normal controls (NC), eleven HLA-A2 restricted healthy individuals, who were negative for HBV markers, were also enrolled. Clinical characteristics of all enrolled subjects were shown in **Table 1**. Blood samples were collected from all enrolled subjects, while fresh HCC specimens and non-tumor site liver specimens were obtained from HCC patients who underwent surgery in The Second Hospital, Jilin University.

**Table 1:** Clinical characteristics of enrolled subjects.

	NC (*n*=11)	CHB (*n*=27)	HCC (*n*=22)
Gender (Male/Female)	7/4	17/10	14/8
Age (Years)	44.5 ± 10.9	42.3 ± 8.7	48.7 ± 14.2
HBV DNA (log_10_ IU/ml)	Not available	5.83 ± 1.16	5.07 ± 1.39
HBsAg positive (*n*)	0	27	22
anti-HBs positive (*n*)	0	0	0
HBeAg positive (*n*)	0	14	10
anti-HBe positive (*n*)	0	13	12
anti-HBc positive (*n*)	0	27	22
ALT (IU/L)	<40 (9~37)	>80 (83~547)	23~446
AFP (ng/mL)	<20 (4.03~11.81)	<200 (7.81~177.4)	>400 (447.2~120500)
BCLC stage (A/B/C/D)	Not available	Not available	7/7/5/3
Cirrhosis (*n*)	Not available	Not available	14

HBsAg, hepatitis B surface antigen; anti-HBs, anti-hepatitis B surface; HBeAg, hepatitis B e antigen; anti-HBe, anti-hepatitis B e; anti-HBc, anti-hepatitis B core; ALT, alanine aminotransferase; AFP, alpha fetoprotein; BCLC, Barcelona Clinic Liver Cancer.

### Isolation of Peripheral Blood Mononuclear Cells and Intrahepatic Lymphocytes

Three milliliter (mL) of coagulant and 20 mL of ethylene diamine tetraacetic acid anticoagulant peripheral bloods were collected from each enrolled subject. PBMCs were isolated by density gradient centrifugation using Ficoll-Hypaque (Sigma-Aldrich, St Louis, MO, USA). IHLs were isolated from liver specimens as previously described (14). Briefly, the liver specimen was diced into small pieces, which was then passed through fine steel sieves. The suspension was incubated at 37°C for 30 min in the presence of collagenase V (0.5 mg/mL) and DNase I (0.001%). 40 mL pre-cold RPMI 1640 was added into suspension, and were centrifuged ay 16 ×*g* for 2 min at 16°C. The supernatant was collected, and was centrifuged at 300 ×*g* for 10 min at 4°C. The IHLs pellet was resuspended in 3 mL sterile 44% Percoll solution in RPMI 1640 (*v*/*v*), and was layered over 5 mL 56% Percoll solution in PBS (*v*/*v*). The mixture was gradiently centrifuged 850 ×*g* for 30 min at 20°C. Interphase, which contained purified IHLs, was harvested.

### Purification of CD8^+^ T Cells and CD4^+^CXCR3^-^CCR4^-^CCR6^-^ Cells

CD8^+^ T cells and CD4^+^ T cells were purified using Human CD8^+^ T Cell Isolation Kit (Miltenyi, Bergisch Gladbach, Germany) and Human CD4^+^ T Cell Isolation Kit (Miltenyi), respectively. CD4^+^ T cells were then stained with CXCR3-PE (BD Bioscience, San Jose, CA, USA), CCR4-PE (BD Bioscience), and CCR6-PE (BD Bioscience). CD4^+^CXCR3^-^CCR4^-^CCR6^-^ cells were negatively selected using FACS LSR II Flow cytometer (BD Bioscience).

### Cell Culture and Stimulation

10^4^ of CD4^+^CXCR3^-^CCR4^-^CCR6^-^ cells were stimulated with recombinant human IL-35 (Peprotech, Rocky Hill, NJ, USA) in the presence of anti-CD3/CD28 (1 μg/mL) or recombinant HBV surface antigen (HBsAg, AbD Serotec, Oxford, United Kingdom; 10 μg/mL) for 24 h. Recombinant IL-35 concentration gradient was set as 50 pg/mL, 500 pg/mL, and 1 ng/mL. In co-culture experiments, CD4^+^CXCR3^-^CCR4^-^CCR6^-^ cells were firstly stimulated with recombinant human IL-35 (Peprotech; 1 ng/mL) (12–14, 16) in the presence of anti-CD3/CD28 (1 μg/mL) for 24 h. Cells were washed twice to remove exogenous IL-35, and then 10^4^ of CD4^+^CXCR3^-^CCR4^-^CCR6^-^ cells were co-cultured with 10^4^ of CD8^+^ T cells in a direct contact manner with 10^5^ of HepG2.2.15 cells, which were also HLA-A2 restricted (17). Anti-CD3/CD28 (1 μg/mL) or HBsAg (10 μg/mL) and HBV core 18-27 epitope (sequence: FLPSDFFPSV; 5 μg/mL) were added for maintenance of T cell activation. In certain experiments, anti-IL-9 neutralization antibody (R&D System, Minneaposlis, MN, USA; 5 μg/mL) was also added for blocking IL-9 activity.

### Enzyme Linked Immunosorbent Assay

Cytokine expression was measured using commercial ELISA kits (CUSABIO, Wuhan, Hubei Province, China), including human IL-35 ELISA kit (Catolog No. CSB-E13126h), human IL-9 ELISA kit (Catolog No. CSB-E04642h), human IFN-γ ELISA kit (Catolog No. CSB-E04577h), and human tumor necrosis factor-α (TNF-α) ELISA kit (Catolog No. CSB-E04740h).

### Flow Cytometry

PBMCs or IHLs were stimulated with either phorbol myristate acetate (PMA) (50 ng/ml)+ionomycin (1 μg/mL) or HBsAg (10 μg/mL) in the presence of brefeldin A (10 μg/mL) for 6 hours. Cells were transferred to FACS tubes, and were stained with anti-CD3-FITC (BD Bioscience, San Jose, CA, USA), anti-CD4-PerCP (BD Bioscience), and anti-CD8-APC (BD Bioscience) for 30 min in the dark at 4 °C. Cells were washed twice, and were stained with anti-IL-9-PE (eBioscience, San Diego, CA, USA) for 30 min at room temperature after fixation and permeabilization. Isotype controls were used to enable correct compensation and confirm antibody specificity. Acquisitions were performed using Cell Quest Pro Software (BD Bioscience Immunocytometry Systems, San Jose, CA, USA) in a FACS Calibur analyzer (BD Bioscience Immunocytometry Systems). Data were analyzed using FlowJo Software Version 10.0 for Windows (Tree Star, Ashland, OR, USA).

### Real-Time PCR

Total RNA was isolated using Trizol reagent (Invitrogen, Carlsbad, CA, USA). cDNA was synthesized with random hexamers using PrimeScript RT Master Mix (TaKaRa, Beijing, China). Real-time PCR was performed using TB Green Premix Ex *Taq* (TaKaRa). The relative gene expression was quantified using 2*^-ΔΔCT^* method with ABI7500 System Sequence Detection software (Applied Biosystems, Foster, CA, USA). The primers sequences for PU.1 and β-actin were shown as following. PU.1 forward: 5’-AGA AGA AGA TCC GCC TGT ACCA-3’, PU.1 reverse: 5’-GTG CTT GGA CGA GAA CTG GAA-3’; β-actin forward: 5’-AGT TGC GTT ACA CCC TTT CTT G-3’, β-actin reverse: 5’-TCACCTTCA CCGTTCCAGTTT-3’ (18). Primes for IL-12 receptor β2 (IL-12Rβ2; Catolog No. qHsaCID0006511) and gp130 (Catolog No. qHsaCID0007540) was obtained from Bio-Rad (Hercules, CA, USA).

### Cytotoxicity of Target Cells

The cytotoxicity of target HepG2.2.15 cells was assessed by measuring lactate dehydrogenase (LDH) expression in the cultured supernatants at the end of incubation period using LDH Cytotoxicity Assay Kit (Beyotime) as previously described (14). LDH expression in HepG2.2.15 cells were determined as low-level control, while LDH expression in Triton X-100-treated HepG2.2.15 cells was determined as high-level control. The percentage of cell death was calculated using the following equation: (experimental value – low-level control)/(high-level control – low-level control) × 100% (14).

### Statistical Analysis

Data were analyzed using SPSS21.0 for Window (SPSS, Chicago, IL, USA). Shapiro-Wilk test was firstly used for normal distribution assay, and all data were following normal distribution. Data were presented as mean ± standard deviation. Statistical significance was determined by one-way analysis of variance (one-way ANOVA), Student’s-Newman-Keuls (SNK)-*q* test, or Student *t* test. All tests were two-tailed, and a *P*-value < 0.05 was considered statistically significant.

## Results

### Serum IL-35 Level Was Up-Regulated but IL-9 Was Down-Regulated in Hepatitis B-Related HCC Patients

**Figure 1:**
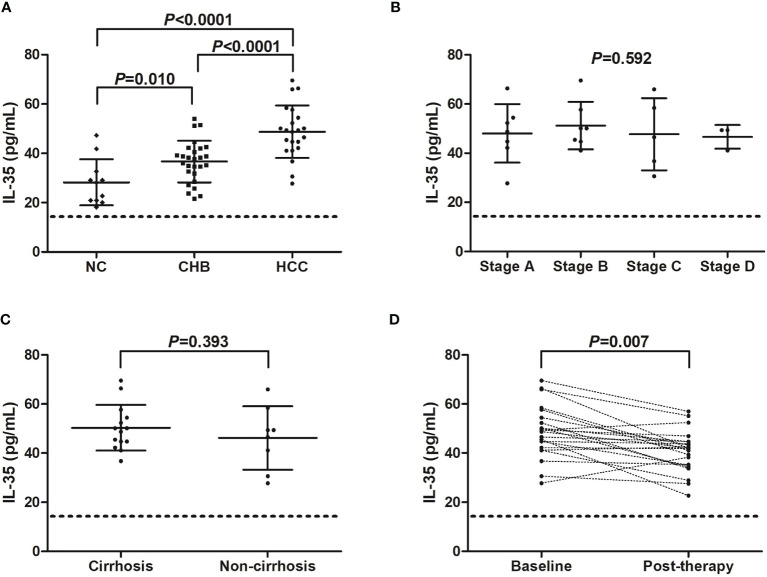
IL-35 level in hepatitis B-related hepatocellular carcinoma (HCC). **(A)** Serum IL-35 concentration was measured by ELISA in normal controls (NC, *n*=11), chronic hepatitis B (CHB) patients (*n*=27), and hepatitis B-related HCC patients (*n*=22). Significance was assessed using one-way ANOVA and SNK-*q* test. **(B)** Serum IL-35 level was compared among hepatitis B-related HCC patients in BCLC stage A (*n*=7), stage B (*n*=7), stage C (*n*=5), and stage D (*n*=3). Significance was assessed using one-way ANOVA. **(C)** Serum IL-35 was also compared between hepatitis B-related HCC patients with cirrhosis (*n*=14) and without cirrhosis (*n*=8). Significance was assessed using Student *t* test. **(D)** Serum IL-35 was also measured in hepatitis B-related HCC patients who underwent hepatic carcinectomy (n=13) or TACE (*n*=9), and was compared between baseline and 2 months post therapy. The dotted line presented lower detection limit for IL-35 (15.6 pg/mL). Significance was assessed using paired *t* test.

We firstly screened the IL-35 and IL-9 level in the serum in hepatitis B-related HCC patients. Serum IL-35 was increasingly expressed in both CHB patients (36.69 ± 8.43 pg/mL), and hepatitis B-related HCC patients (48.79 ± 10.65 pg/mL) compared with in NC (28.26 ± 9.36 pg/mL) (*P*<0.05, SNK-*q* tests, **Figure 1A**). Importantly, serum IL-35 level was also elevated in hepatitis B-related HCC patients compared with in CHB patients (*P*<0.0001, SNK-*q* test, **Figure 1A**). There were no significant differences of serum IL-35 level among hepatitis B-related HCC patients with different stages (*P*=0.592, one-way ANOVA, **Figure 1B**). IL-35 level in the serum also did not reveal statistical difference between HCC patients with cirrhosis and HCC patients without cirrhosis (50.30 ± 9.31 pg/mL *vs* 46.15 ± 12.92 pg/mL, *P*=0.393, Student *t* test, **Figure 1C**). Thirteen HCC patients (seven in stage A and six in stage B) underwent hepatic carcinectomy, while other nine patients (one in stage B, five in stage C, and three in stage D) underwent transcatheter arterial chemoembolization (TACE). Serum samples were collected 2 months post therapy. There was a significant decreasing trend of serum IL-35 post-therapy in hepatitis B-related HCC patients (40.57 ± 8.44 pg/mL *vs* 48.79 ± 10.65 pg/mL, *P*=0.007, paired *t* test, **Figure 1D**).

**Figure 2:**
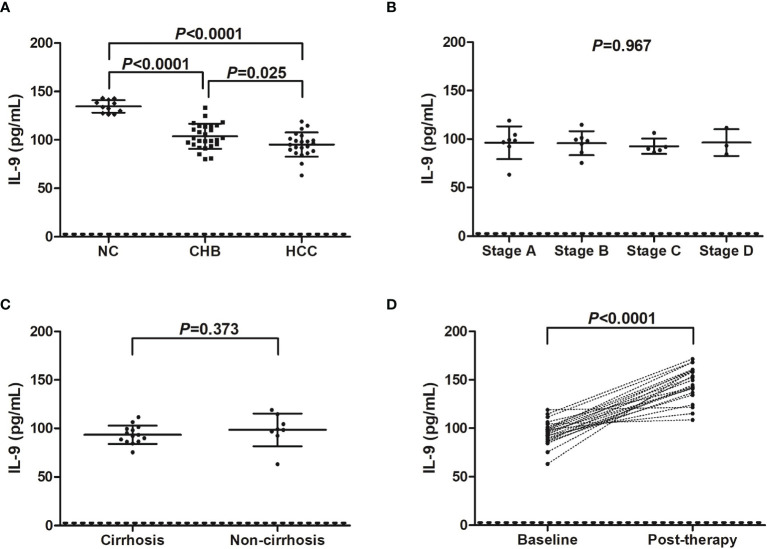
IL-9 level in hepatitis B-related hepatocellular carcinoma (HCC). **(A)** Serum IL-9 concentration was measured by ELISA in normal controls (NC, *n*=11), chronic hepatitis B (CHB) patients (*n*=27), and hepatitis B-related HCC patients (*n*=22). Significance was assessed using one-way ANOVA and SNK-*q* test. **(B)** Serum IL-9 level was compared among hepatitis B-related HCC patients in BCLC stage A (*n*=7), stage B (*n*=7), stage C (*n*=5), and stage D (*n*=3). Significance was assessed using one-way ANOVA. **(C)** Serum IL-9 was also compared between hepatitis B-related HCC patients with cirrhosis (*n*=14) and without cirrhosis (*n*=8). Significance was assessed using Student *t* test. **(D)** Serum IL-9 was also measured in hepatitis B-related HCC patients who underwent hepatic carcinectomy (n=13) or TACE (*n*=9), and was compared between baseline and 2 months post therapy. The dotted line presented lower detection limit for IL-9 (3.9 pg/mL). Significance was assessed using paired *t* test.

Serum IL-9 level was notably reduced in both CHB patients (103.8 ± 12.95 pg/mL), and hepatitis B-related HCC patients (95.27 ± 12.53 pg/mL) compared with in NC (134.6 ± 6.49 pg/mL) (*P*<0.0001, SNK-*q* tests, **Figure 2A**). IL-9 expression in the serum was also significantly decreased in hepatitis B-related HCC patients compared with in CHB patients (*P*=0.025, SNK-*q* test, **Figure 2A**). Furthermore, serum IL-9 level did not show remarkable differences among HCC patients with different stages (*P*=0.967, one-way ANOVA, **Figure 2B**). There was no statistical difference of serum IL-9 level between HCC patients with and without cirrhosis (93.42 ± 9.46 pg/mL *vs* 98.50 ± 16.92 pg/mL, *P*=0.373, Student *t* test, **Figure 2C**). Importantly, serum IL-9 level was robustly increased in HCC patients post therapy (146.2 ± 17.43 pg/mL *vs* 95.27 ± 12.53 pg/mL, *P*<0.0001, paired *t* test, **Figure 2D**). However, there was no significant correlation between IL-35 and IL-9 level in either group (*P*>0.05).

### Non-Specific and HBV-Specific Th9 Cells Was Down-Regulated in CHB and Hepatitis B-Related HCC Patients

**Figure 3:**
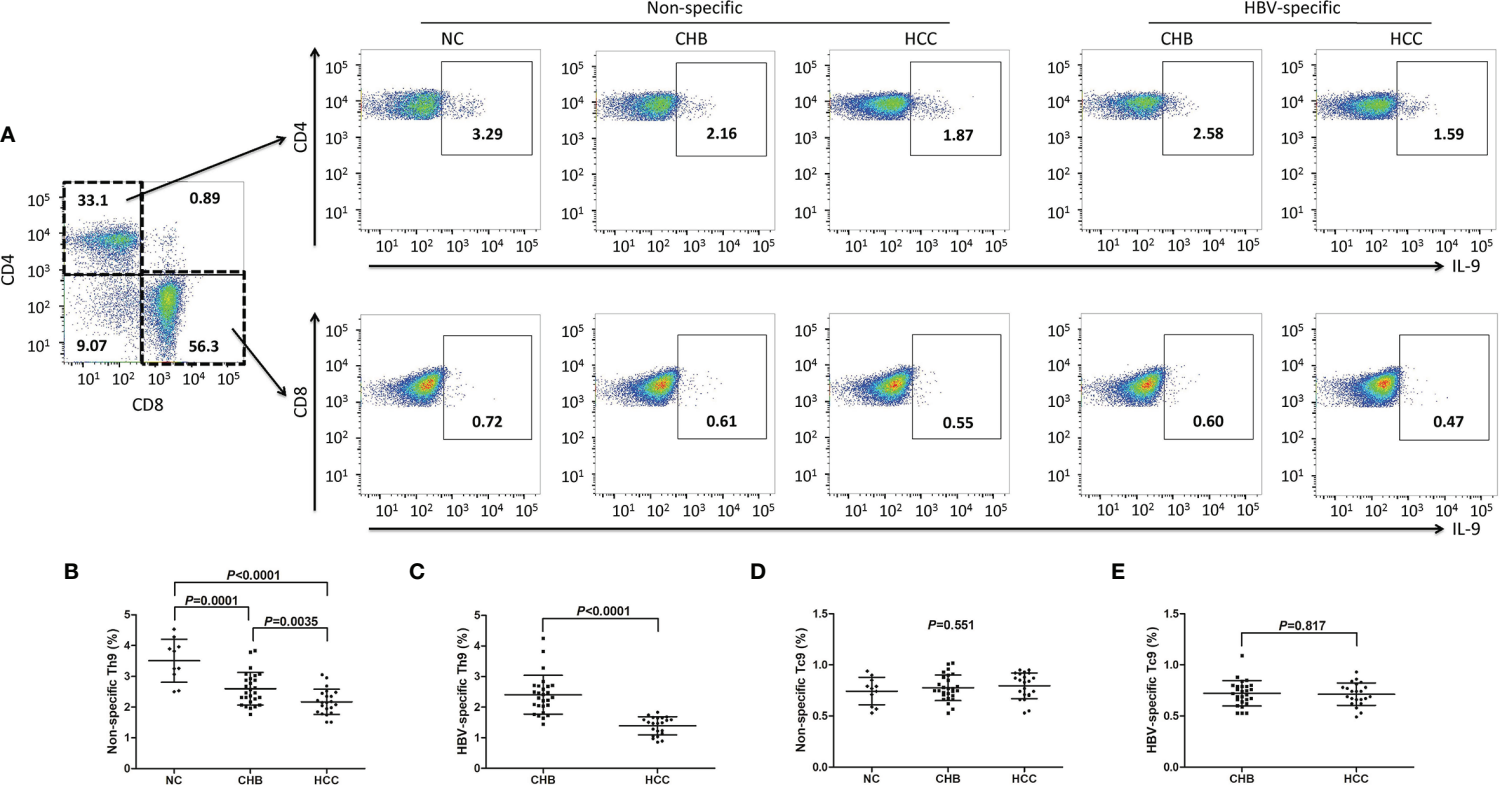
Peripheral non-specific and HBV-specific Th9/Tc9 cells in hepatitis B-related hepatocellular carcinoma (HCC). **(A)** Peripheral blood mononuclear cells (PBMCs) were isolated from all enrolled subjects, including normal controls (NC, n=11), chronic hepatitis B (CHB) patients (*n*=27), and hepatitis B-related HCC patients (*n*=22). PBMCs were stimulated with either phorbol myristate acetate (50 ng/ml)+ionomycin (1 μg/ml) (for non-specific stimulation) or HBsAg (for HBV-specific stimulation) in the presence of brefeldin A (10 μg/ml) for 6 hours, and were then stained with anti-CD3, anti-CD4, anti-CD8, and anti-IL-9. CD3^+^CD4^+^IL-9^+^ Th9 cells and CD3^+^CD8^+^IL-9^+^ Tc9 cells were analyzed by flow cytometry. The representative flow dots for non-specific and HBV-specific Th9 and Tc9 cells in NC, CHB, and hepatitis B-related HCC patients were shown. **(B)** The percentage of peripheral non-specific Th9 cells was compared among NC, CHB, and hepatitis B-related HCC patients. Significance was assessed using one-way ANOVA and SNK-*q* test. **(C)** The percentage of peripheral HBV-specific Th9 cells was compared between CHB and hepatitis B-related HCC patients. Significance was assessed using Student *t* test. **(D)** The percentage of peripheral non-specific Tc9 cells was compared among NC, CHB, and hepatitis B-related HCC patients. Significance was assessed using one-way ANOVA and SNK-*q* test. **(E)** The percentage of peripheral HBV-specific Tc9 cells was compared between CHB and hepatitis B-related HCC patients. Significance was assessed using Student *t* test.

PBMCs from all enrolled subjects were stimulated with either PMA+ionomycin (non-specific stimulation) or HBsAg (HBV-specific stimulation). Cells were then stained with anti-CD3, anti-CD4, anti-CD8, and anti-IL-9. CD3^+^CD4^+^IL-9^+^ Th9 cells and CD3^+^CD8^+^IL-9^+^ Tc9 cells were analyzed by flow cytometry. There were no significant differences of either CD4^+^CD3^+^ or CD8^+^CD3^+^ T cell percentage within CD3^+^ T cells in PBMCs among groups (*P*>0.05, one-way ANOVA, **Figures S1A, B**). The representative flow dots for peripheral non-specific and HBV-specific Th9 and Tc9 cells were shown in **Figure 3A**. The percentage of peripheral non-specific Th9 cells was significantly down-regulated in CHB patients (2.60 ± 0.53%) and hepatitis B-related HCC patients (2.17 ± 0.41%) compared with in NC (3.51 ± 0.70%) (*P*<0.001, SNK-*q* tests, **Figure 3B**). Non-specific Th9 cells frequency was also remarkably reduced in hepatitis B-related HCC patients compared with in CHB patients (*P*=0.0035, SNK-*q* test, **Figure 3B**). Importantly, HBV-specific Th9 cells was also notably decreased in hepatitis B-related HCC patients (1.39 ± 0.30%) compared with in CHB patients (2.40 ± 0.64%) (*P*<0.0001, Student *t* test, **Figure 3C**). Moreover, there was no statistical difference of peripheral non-specific Tc9 cells among NC (0.74 ± 0.13%), CHB patients (0.78 ± 0.13%), and hepatitis B-related HCC patients (0.79 ± 0.12%) (*P*=0.551, one-way ANOVA, **Figure 3D**). There was also no significant difference of peripheral HBV-specific Tc9 cells between CHB and hepatitis B-related HCC patients (0.72 ± 0.12% *vs* 0.71 ± 0.11%, *P*=0.817, Student *t* test, **Figure 3E**).

**Figure 4:**
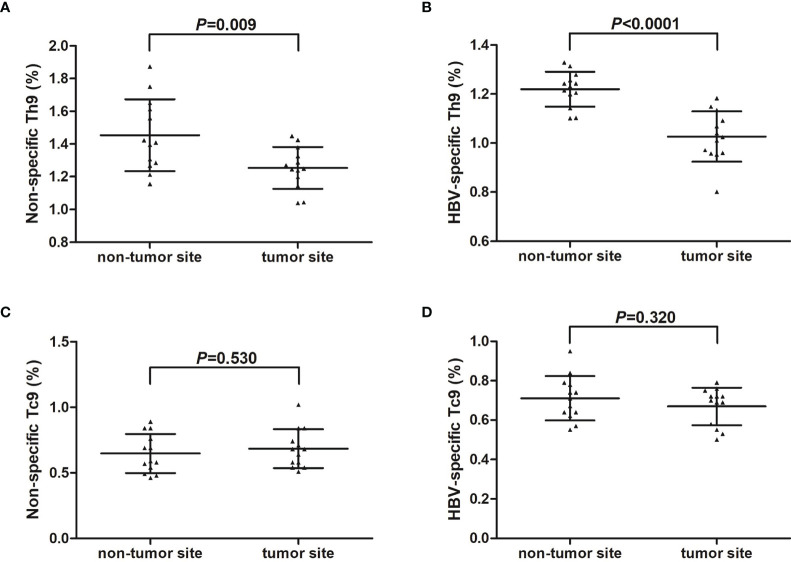
Liver-infiltrating non-specific and HBV core-specific Th9/Tc9 cells in hepatitis B-related hepatocellular carcinoma (HCC). Intrahepatic lymphocytes (IHLs) were isolated from fresh HCC specimens and non-tumor site liver specimens in thirteen HCC patients (seven in stage A and six in stage B) who underwent hepatic carcinectomy. IHLs were stimulated with either phorbol myristate acetate (50 ng/ml)+ionomycin (1 μg/ml) (for non-specific stimulation) or HBsAg (for HBV-specific stimulation) in the presence of brefeldin A (10 μg/ml) for 6 hours, and were then stained with anti-CD3, anti-CD4, anti-CD8, and anti-IL-9. CD3^+^CD4^+^IL-9^+^ Th9 cells and CD3^+^CD8^+^IL-9^+^ Tc9 cells were analyzed by flow cytometry. **(A)** The percentage of liver-infiltrating non-specific Th9 cells was compared between non-tumor site and tumor site. Significance was assessed using Student *t* test. **(B)** The percentage of liver-infiltrating HBV-specific Th9 cells was compared between non-tumor site and tumor site. Significance was assessed using Student *t* test. **(C)** The percentage of liver-infiltrating non-specific Tc9 cells was compared between non-tumor site and tumor site. Significance was assessed using Student *t* test. **(D)** The percentage of liver-infiltrating HBV-specific Tc9 cells was compared between non-tumor site and tumor site. Significance was assessed using Student *t* test.

IHLs were isolated from fresh HCC specimens and non-tumor site liver specimens in thirteen HCC patients (seven in stage A and six in stage B) who underwent hepatic carcinectomy. IHLs were stimulated in both non-specific and HBV-specific manners, and were then stained with anti-CD3, anti-CD4, anti-CD8, and anti-IL-9. Th9 cells and Tc9 cells were analyzed by flow cytometry. There were no significant differences of either CD4^+^CD3^+^ or CD8^+^CD3^+^ T cell percentage within CD3^+^ T cells in IHLs between non-tumor site and tumor site (*P*>0.05, Student *t* test, **Figures S1C, D**). The percentage of liver-infiltrating non-specific Th9 cells was significantly down-regulated in tumor site (1.25 ± 0.13%) compared with in non-tumor site (1.45 ± 0.22%) (*P*=0.009, Student *t* test, **Figure 4A**). The percentage of liver-infiltrating HBV-specific Th9 cells was also notably reduced in tumor site (1.03 ± 0.10%) compared with in non-tumor site (1.22 ± 0.07%) (*P*<0.0001, Student *t* test, **Figure 4B**). However, there was no remarkable difference of either liver-infiltrating non-specific Tc9 cells or HBV-specific Tc9 cells between non-tumor and tumor site (*P*>0.05, Student *t* tests, **Figures 4C, D**).

### Th9 Cells Promoted Cytotoxicity of Non-Specific and HBV-Specific CD8^+^ T Cells *via* IL-9 Secretion

**Figure 5:**
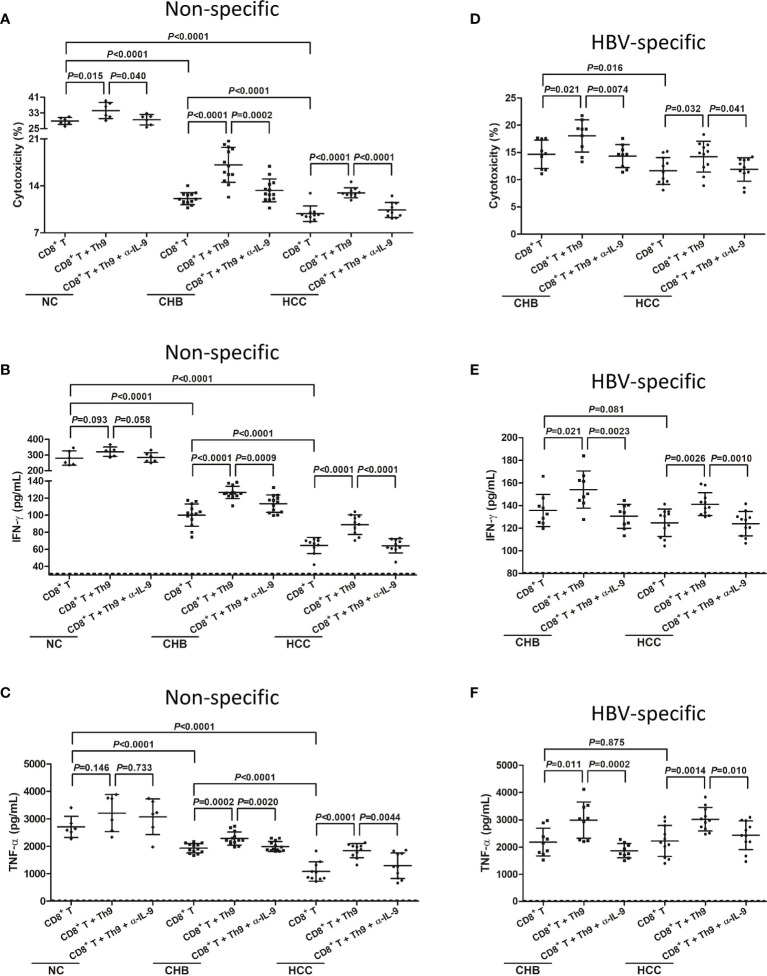
Influence of Th9 cells to non-specific and HBV-specific CD8^+^ T cells in hepatitis B-related hepatocellular carcinoma (HCC). 10^4^ of peripheral CD8^+^ T cells from normal controls (NC, *n*=6), chronic hepatitis B (CHB) patients (*n*=13), and hepatitis B-related HCC patients (*n*=10) were co-cultured with HepG2.2.15 cells in a direct contact manner in the presence or absence of 10^4^ of autologous Th9 cells or anti-IL-9 neutralization antibody (5 μg/mL). Anti-CD3/CD28 (1 μg/mL) was added for maintenance of non-specific T cell response. Supernatants were harvested 48 hours post co-culture. **(A)** Percentage of target HepG2.2.15 cell death was measured by LDH release, and was compared among groups in non-specific manners. Significance was assessed using one-way ANOVA and SNK-*q* test. **(B)** IFN-γ and **(C)** TNF-α, which secreted by non-specific CD8^+^ T cells, was measured by ELISA, and was compared among groups. Significance was assessed using one-way ANOVA and SNK-*q* test. 10^4^ of peripheral CD8^+^ T cells from CHB patients (*n*=9), and hepatitis B-related HCC patients (*n*=11) were co-cultured with HepG2.2.15 cells in a direct contact manner in the presence or absence of 10^4^ of autologous Th9 cells or anti-IL-9 neutralization antibody (5 μg/mL). HBV core 18-27 epitope (sequence: FLPSDFFPSV; 5 μg/mL) was added for maintenance of HBV-specific T cell response. Supernatants were harvested 48 hours post co-culture. **(D)** Percentage of target HepG2.2.15 cell death was measured by LDH release, and was compared among groups in HBV core-specific manners. Significance was assessed using one-way ANOVA and SNK-*q* test. **(E)** IFN-γ and **(F)** TNF-α, which secreted by HBV core-specific CD8^+^ T cells, was measured by ELISA, and was compared among groups. The dotted line presented lower detection limit for IFN-γ (1.56 pg/mL) and TNF-α (1.95 pg/mL), respectively. Significance was assessed using one-way ANOVA and SNK-*q* test.

mRNA expression of Th9 transcription factor PU.1 was more than 10-fold elevation in CD4^+^CXCR3^-^CCR4^-^CCR6^-^ cells compared with in CD4^+^CXCR3^+^CCR4^+^CCR6^+^ cells (*P*<0.0001, Student *t* test, **Figure S2**), indicating that CD4^+^CXCR3^-^CCR4^-^CCR6^-^ cells were mostly Th9 cells (19, 20). Th9 cells did not reveal cytotoxicity to HepG2.2.15 cells, as the percentage of target cell death induced by Th9 cells was less than 1% (data not shown). Peripheral CD8^+^ T cells from six NC, thirteen CHB, and ten hepatitis B-related HCC patients were co-cultured with HepG2.2.15 cells in the presence or absence of autologous Th9 cells and anti-IL-9 neutralization antibody. Anti-CD3/CD28 was added for maintenance of non-specific T cell response. Supernatants were harvested 48 hours post co-culture. Non-specific CD8^+^ T cells from NC revealed stronger cytotoxicity than from CHB patients and hepatitis B-related HCC patients, which presented as elevated induction of target HepG2.2.15 cell death (28.88 ± 1.80%; *P*<0.0001, SNK-*q* tests, **Figure 5A**). The cytotoxicity of peripheral non-specific CD8^+^ T cells was also reduced in hepatitis B-related HCC patients (9.85 ± 1.16%) compared with in CHB patients (12.10 ± 0.90%) (*P*<0.0001, SNK-*q* tests, **Figure 5A**). Autologous Th9 cells enhanced the cytotoxicity of CD8^+^ T cells to HepG2.2.15 cells *in vitro* in all three groups (*P*<0.05, SNK-*q* tests, **Figure 5A**). Importantly, administration of anti-IL-9 neutralization antibody dampened the enhancement of Th9-induced cytotoxicity of peripheral non-specific CD8^+^ T cells in all three groups (*P*<0.05, SNK-*q* tests, **Figure 5A**). Moreover, IFN-γ and TNF-α level in the supernatant was significantly higher in CD8^+^ T cells from NC compared with CHB and hepatitis B-related HCC patients (*P*<0.0001, SNK-*q* tests, **Figures 5B, C**). Either Th9 cells co-culture or anti-IL-9 neutralization antibody administration did not affect non-specific CD8^+^ T cell-induced IFN-γ or TNF-α secretion in NC (*P*>0.05, SNK-*q* tests, **Figures 5B, C**). In contrast, Th9 cells co-culture remarkably promoted IFN-γ and TNF-α secretion by CD8^+^ T cells from CHB and hepatitis B-related HCC patients (*P*<0.001, SNK-*q* tests, **Figures 5B, C**), while anti-IL-9 neutralization antibody administration dampened Th9-mediated elevation of IFN-γ and TNF-α production by CD8^+^ T cells in CHB and hepatitis B-related HCC patients (*P*<0.01, SNK-*q* tests, **Figures 5B, C**).

Furthermore, peripheral CD8^+^ T cells from nine CHB and eleven hepatitis B-related HCC patients were co-cultured with HepG2.215 cells in the presence of HBV core 18-27 peptide for maintenance of HBV-specific T cell response. Th9 cells and/or anti-IL-9 neutralization antibody were added for stimulation. HBV-specific CD8^+^ T cells from CHB patients exhibited stronger cytotoxicity than from hepatitis B-related HCC patients, which presented as higher percentage of HepG2.2.15 cell death (14.67 ± 2.62% *vs* 11.63 ± 2.46%, *P*=0.016, SNK-*q* test, **Figure 5D**). However, there were no remarkable differences of secreted IFN-γ or TNF-α by CD8^+^ T cells between CHB and hepatitis B-related HCC patients (*P*>0.05, SNK-*q* tests, **Figures 5E, F**). Th9 co-culture enhanced HBV-specific CD8^+^ T cell-induced target cell death and cytokine production in both CHB and hepatitis B-related HCC patients (*P*<0.05, SNK-*q* tests, **Figures 5D–F**). Administration of anti-IL-9 neutralization antibody blocked Th9-induced CD8^+^ T cells cytotoxicity in both CHB and hepatitis B-related HCC patients (*P*<0.05, SNK-*q* tests, **Figures 5D–F**).

### IL-35 Stimulation Suppressed Non-Specific and HBV-Specific Th9 Cells in CHB and Hepatitis B-Related HCC Patients

**Figure 6:**
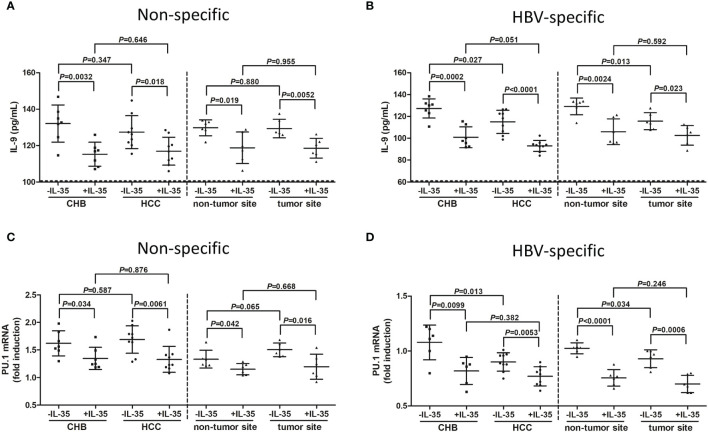
Influence of recombinant IL-35 to non-specific and HBV-specific Th9 cells in hepatitis B-related hepatocellular carcinoma (HCC). CD4^+^CXCR3^-^CCR4^-^CCR6^-^ cells were purified from peripheral blood mononuclear cells (PBMCs) of chronic hepatitis B (CHB) patients (*n*=7) and hepatitis B-related HCC patients (*n*=9), as well as from intrahepatic lymphocytes (IHLs) of fresh HCC specimens and non-tumor site liver specimens in HCC patients (*n*=6). 10^4^ of CD4^+^CXCR3^-^CCR4^-^CCR6^-^ cells were stimulated with either anti-CD3/CD28 (1 μg/mL) or HBsAg (10 μg/mL) in the presence or absence of recombinant IL-35 (1 ng/mL) for 24 hours. Supernatants and cells were harvested. IL-9 level in the supernatants was measured by ELISA, while PU.1 mRNA expression in Th9 cells was quantified by real-time PCR. **(A)** IL-9 secretion by non-specific Th9 cells was compared among groups with or without IL-35 stimulation. Significance was assessed using one-way ANOVA and SNK-*q* test. **(B)** IL-9 secretion by HBV-specific Th9 cells was compared among groups with or without IL-35 stimulation. Significance was assessed using one-way ANOVA and SNK-*q* test. **(C)** PU.1 mRNA expression in non-specific Th9 cells was compared among groups with or without IL-35 stimulation. Significance was assessed using one-way ANOVA and SNK-*q* test. **(D)** PU.1 mRNA expression in HBV-specific Th9 cells was compared among groups with or without IL-35 stimulation. The dotted line presented lower detection limit for IL-9 (3.9 pg/mL). Significance was assessed using one-way ANOVA and SNK-*q* test.

mRNA of IL-35 receptor subsets, including IL-12Rβ2 and gp130, could be detected in CD4^+^CXCR3^-^CCR4^-^CCR6^-^ Th9 cells (**Figure S3**). There was no significant difference of either IL-12Rβ2 or gp130 mRNA expression among NC, CHB patient, and hepatitis B-related HCC patients (**Figure S3**). Moreover, 1 ng/mL of IL-35 suppressed cellular proliferation (**Figure S4**) and only 1 ng/mL of IL-35 reduced signal transducer and activator of transcription 1 (STAT1) and STAT3 phosphorylation (**Figure S5**), confirming the immunosuppressive activity of IL-35 at that concentration. CD4^+^CXCR3^-^CCR4^-^CCR6^-^ Th9 cells were purified from PBMCs of seven CHB patients and nine hepatitis B-related HCC patients, as well as from IHLs of six hepatitis B-related HCC patients (HCC specimens and non-tumor site liver specimens). Th9 cells were stimulated with either anti-CD3/CD28 or HBsAg in the presence or absence of recombinant IL-35 for 24 hours. There was no significant difference of IL-9 secretion by peripheral non-specific Th9 cells between CHB patients and hepatitis B-related HCC patients (*P*=0.347, SNK-*q* test, **Figure 6A**), or by liver-infiltrating non-specific Th9 cells between non-tumor site and tumor site (*P*=0.880, SNK-*q* test, **Figure 6A**). However, IL-9 production was notably reduced by peripheral HBV-specific Th9 cells in hepatitis B-related HCC patients compared with in CHB patients (*P*=0.027, SNK-*q* test, **Figure 6B**). IL-9 level was also significantly down-regulated by liver-infiltrating HBV-specific Th9 cells in tumor site compared with in non-tumor site (*P*=0.013, SNK-*q* test, **Figure 6B**). Recombinant IL-35 stimulation remarkably decreased IL-9 production by non-specific and HBV-specific Th9 cells (*P*<0.05, SNK-*q* tests, **Figures 6A, B**). Similarly, there was no statistical difference of PU.1 mRNA expression in peripheral non-specific Th9 cells between CHB patients and hepatitis B-related HCC patients (*P*=0.587, SNK-*q* test, **Figure 6C**), or in liver-infiltrating non-specific Th9 cells between non-tumor site and tumor site (*P*=0.065, SNK-*q* test, **Figure 6C**). PU.1 mRNA expression was down-regulated in peripheral non-specific Th9 cells in hepatitis B-related HCC patients compared with in CHB patients (*P*=0.0099, SNK-*q* test, **Figure 6D**). PU.1 mRNA expression was also reduced in liver-infiltrating HBV-specific Th9 cells in tumor site compared with in non-tumor site (*P*=0.034, SNK-*q* test, **Figure 6D**). Recombinant IL-35 stimulation also significantly decreased PU.1 mRNA expression in non-specific and HBV-specific Th9 cells (*P*<0.05, SNK-*q* tests, **Figures 6C, D**).

### IL-35 Stimulation Dampened Th9-Induced CD8^+^ T Cell Cytotoxicity in CHB and Hepatitis B-Related HCC Patients

**Figure 7:**
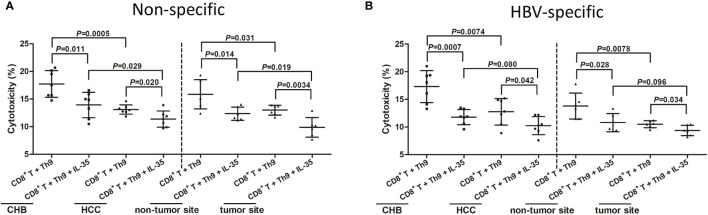
Influence of recombinant IL-35 to Th9-induced CD8^+^ T cell cytotoxicity in hepatitis B-related hepatocellular carcinoma (HCC). CD4^+^CXCR3^-^CCR4^-^CCR6^-^ cells were purified from peripheral blood mononuclear cells (PBMCs) of chronic hepatitis B (CHB) patients (*n*=7) and hepatitis B-related HCC patients (*n*=7), as well as from intrahepatic lymphocytes (IHLs) of fresh HCC specimens and non-tumor site liver specimens in HCC patients (*n*=6). CD4^+^CXCR3^-^CCR4^-^CCR6^-^ Th9 cells were stimulated with anti-CD3/CD28 (1 μg/mL) or HBsAg (10 μg/mL) in the presence or absence of recombinant IL-35 (1 ng/mL) for 24 hours. 10^4^ of stimulated Th9 cells were co-cultured with 10^4^ of autologous CD8^+^ T cells in a direct contact manner with 10^5^ of HepG2.2.15 cells in the presence of anti-CD3/CD28 (1 μg/mL) or HBV core 18-27 peptide (5 μg/mL). Supernatants were harvested 48 hours post co-culture. **(A)** Percentage of target HepG2.2.15 cell death was measured by LDH release, and was compared among groups with or without IL-35 stimulation in non-specific manners. Significance was assessed using one-way ANOVA and SNK-*q* test. **(B)** Percentage of target HepG2.2.15 cell death was measured by LDH release, and was compared among groups with or without IL-35 stimulation in HBV-specific manners. Significance was assessed using one-way ANOVA and SNK-*q* test.

CD4^+^CXCR3^-^CCR4^-^CCR6^-^ Th9 cells were purified from PBMCs of seven CHB patients and seven hepatitis B-related HCC patients, as well as from IHLs of six hepatitis B-related HCC patients (HCC specimens and non-tumor site liver specimens). Th9 cells were firstly stimulated with either anti-CD3/CD28 (non-specific manner) or HBsAg (HBV-specific manner) in the presence or absence of recombinant IL-35 for 24 hours. Cells were washed twice, and then Th9 cells were co-cultured with autologous CD8^+^ T cells in a direct contact manner with HepG2.2.15 cells in the presence of anti-CD3/CD28 (non-specific manner) or HBV core 18-27 peptide (HBV-specific manner) for another 48 hours. Th9 cells from hepatitis B-related HCC patients or tumor site induced decreased CD8^+^ T cell cytotoxicity than those from CHB patients or non-tumor site in both non-specific and HBV-specific manner (*P*<0.05, SNK-*q* tests, **Figures 7A, B**). Recombinant IL-35 stimulation significantly suppressed Th9-induced CD8^+^ T cell in all groups (*P*<0.05, SNK-*q* tests, **Figures 7A, B**).

## Discussion

IL-9 is a pleiotropic cytokine, which is involved in both immunopathologies and protective immunity (21), produced by a wide variety of cells including mast cells, natural killer T cells, innate lymphoid cells type 2, Th9 cells, and Tc9 cells (22). Th9 and Tc9 cells are considered to the main T cells that produce IL-9. Increased expression of IL-9 was found in acute HIV infection, while decreased IL-9 level in chronic HIV infection (23). Th9 and Tc9-secreting IL-9 still strongly inhibited coxsackie virus B3 replication without induction of apoptosis in cardiomyocytes (24), although the viral myocarditis did not mainly contribute to differentiation and proliferation of Th9 cells (25). In line with previous report (9), our current results suggested a decreased serum IL-9 and circulating non-specific and HBV-specific Th9 cells, but not Tc9 cells, in CHB and hepatitis B-related HCC. This indicated that decreased Th9 cells might mainly account for down-regulation of IL-9 during chronic HBV infection. However, Th9 cells-secreting IL-9 yielded different responses depending on types of cancers (26). In cervical cancer, IL-9 enhanced anti-tumor response through T cell cytotoxicity and controlled malignant cell transformation (27). In contrast, in lung cancer, tumor-infiltrating Th9 cells promoted migration and induced metastatic spreading (28). Herein, we found that hepatitis B-related HCC patients even had lower serum IL-9 and peripheral Th9 cells than CHB patients. Importantly, liver-infiltrating Th9 cells, but not Tc9 cells, was also decreased in tumor sites compared with in non-tumor sites in both non-specific and HBV-specific manner, suggesting that depletion of Th9 response might also one of the hallmarks in HBV-induced immune dysfunction or exhaustion. Increased IL-9 and decreased IL-35 level in hepatitis B-related HCC patients post therapy might partly reflect the restoration of effort T cell function. However, we did not find remarkable correlation between IL-9 and IL-35 expression. This might be due to the limited sample size and the indirect regulation of IL-35 to IL-9 production. Moreover, there was no significant difference IL-9 level secreted from non-specific Th9 cells between CHB and hepatitis B-related HCC patients. However, the percentage of non-specific and HBV-specific Th9 cells was down-regulated in hepatitis B-related HCC patients. This might be partly due to the difference in expression of precursor molecules, including cytokines and chemokines, of this subpopulation. Our current results were not fully consistent with the study by Tan et al. (29). They found the elevation of Th9 cells infiltration in peritumor and tumor sites in HCC, and higher tumor-infiltrating Th9 cells indicated a decreased survival period (29). This controversy might be partly because of the different immune status between two studied populations. We only enrolled hepatitis B-related HCC, while Tan et al. enrolled HCC patients with various underlying liver diseases (29). Due to the dual natural of IL-9 in viral infection and cancers, further experiments were needed for elucidation of Th9 activity in chronic HBV-infected diseases, including CHB and HCC.

Our previous reports demonstrated peripheral and liver-infiltrating CD8^+^ T cell exhaustion in chronic hepatitis B (12), non-viral hepatitis-related HCC (14), and viral hepatitis-related acute-on-chronic liver failure (16). The current *in vitro* data also supported that peripheral CD8^+^ T cells from hepatitis B-related HCC patients revealed CD8^+^ T cell dysfunction in both non-specific and HBV-specific manner. Similarly, liver-infiltrating non-specific and HBV-specific CD8^+^ T cells from tumor sites also showed exhausted phenotype, which presented as decreased cytotoxicity to target HBV infected HCC cell line. Th9 cells have been shown to enhanced the cytotoxicity of peripheral non-specific CD8^+^ T cells in a direct cell-to-cell contact manner in acute coronary syndrome (19) and breast cancer (20). Our present results also showed that Th9 cells promoted CD8^+^ T cell cytotoxicity in both non-specific and HBV-specific condition, further confirming the regulatory property of Th9 cells to CD8^+^ T cells. Importantly, blockade of IL-9 notably diminished Th9-mediated enhancement of CD8^+^ T cell cytotoxicity, indicating that IL-9 was one of the major effector cytokine for immunomodulatory activity of Th9 cells (19). However, Th9 cells revealed dual effects in tumor development (30). Th9 cells and secreting IL-9 contributed to the functional regulation of mast cells and regulatory T cells, which have been demonstrated to inhibit immune response and promote tumor development in hematological malignancies  (31, 32). In contrast, antigen-specific Th9 cells were effective in eliciting anti-tumor immune responses and suppressing tumor growth in several different solid malignancies (33, 34). Thus, down-regulation of HBV-specific Th9 cells and IL-9 secretion might be insufficient for maintaining CD8^+^ T cells, leading to HBV-specific CD8^+^ T cell dysfunction or exhaustion during chronic HBV infection, especially in hepatitis B-related HCC.

The regulation of antigen-specific Th9 proliferation and differentiation was not fully elucidated. The development of Th9 cells relies on various signaling pathways (30, 35). Several cytokines, including IL-1, IL-7, IL-21, and IL-25, were identified to synergistically promote Th9 cell development (30, 36). In contrast, IFN-γ inhibited Th9 differentiation through IL-27 derived from dendritic cells (37). IL-35 has been proven as an immunosuppressive cytokine through suppression of CD8^+^ T cell cytotoxicity in viral hepatitis and HCC (12, 14). However, a more recent study reported the crosstalk between IL-35-secreting and Th9 cells in IgG4-related disease (15). They found elevations of IL-35-positive cells in liver and pancreatic tissue in IgG4-related disease. Administration of IL-35 enhanced naïve CD4^+^ T cell differentiating towards Th9 cells, leading to the plasma cell differentiation and IgG4 production (15). However, the direct regulatory function of IL-35 to Th9 cells was still unknown. Herein, we showed that IL-35 receptor subunits, IL-12Rβ2 and gp130, could be detected in Th9 cells, indicating the potential direct immunomodulatory activity of IL-35 to Th9 cells. Recombinant IL-35 stimulation to Th9 cells inhibited IL-9 secretion and PU.1 mRNA expression in both non-specific and HBV-specific manner. Importantly, IL-35 administration strongly dampened Th9-induced elevation of non-specific and HBV-specific CD8^+^ T cells in CHB and hepatitis B-related HCC patients. Although we did not find correlation between IL-35 and IL-9 production in CHB or hepatitis B-related HCC patients, our current data still suggested that elevation of IL-35 suppresses HBV-specific Th9 activity during chronic HBV infection. Thus, IL-35 not only eliminated the function of CD8^+^ T cells in a direct way (12, 14, 16), but also reduced IL-9 production by CD4^+^ T cells, and in turn, would lead to a suppression in CD8^+^ T cell-mediated cytotoxicity in hepatitis and HCC patients. The contrary role of IL-35 in regulation of Th9 cell function might be due to the different immune status between autoimmune disorder and chronic infection. Herein, we purified Th9 subset by using a positive selection for CD4 and a negative selection for CXCR3, CCR4, and CCR6 (19, 20). However, there was also a few Th9 cells with CD4^+^CXCR3^+^CCR4^-^CCR6^+^ phenotype has been reported (30, 38, 39). Although we have confirmed PU.1 mRNA expression within CD4^+^CXCR3^-^CCR4^-^CCR6^-^ cells to confirm the Th9 subset, it is not sure how many other Th9 cells were excluded by the current isolation strategy nor if they would have a different type of response to IL-35. However, we cannot directly select IL-9-producing CD4^+^ T cells based on IL-9 staining. Currently, CD4^+^CXCR3^-^CCR4^-^CCR6^-^ phenotype should be the best way for Th9 cell purification.

In summary, elevated IL-35 expression in hepatitis B-related HCC might suppress peripheral and liver-infiltrating antigen-specific Th9 cell differentiation and IL-9 production. Decreased HBV-specific Th9 cells might be insufficient to maintain functional CD8^+^ T cells, which accelerates immune exhaustion in hepatitis-B related HCC. The crosstalk between IL-35 and Th9 cells might be considered as the therapeutic targets for hepatitis B-related HCC.

## Data Availability Statement

The raw data supporting the conclusions of this article will be made available by the authors, without undue reservation.

## Ethics Statement

The studies involving human participants were reviewed and approved by Ethics Committee of The Second Hospital of Jilin University. The patients/participants provided their written informed consent to participate in this study.

## Author Contributions

QZ, LY, and SL performed the study. QZ, LY, SL, and MZ enrolled the patients. QZ, LY, SL, MZ, and ZJ analyzed the data, and prepared the manuscript. ZJ designed and supervised the study. All authors contributed to the article and approved the submitted version.

## Funding

This work was supported by the grant from Natural Science Foundation of Jilin Province (2019021047JC).

## Conflict of Interest

The authors declare that the research was conducted in the absence of any commercial or financial relationships that could be construed as a potential conflict of interest.
