# Supplementary material for: Interleukin-35 Suppresses Interleukin-9-Secreting CD4+ T Cell Activity in Patients With Hepatitis B-Related Hepatocellular Carcinoma
Source: Front Immunol. 2021 Jun 10;12:645835. doi: 10.3389/fimmu.2021.645835 (PMC8222719; doi:10.3389/fimmu.2021.645835)
Supplement: Supplementary file 1 [file DataSheet_1.pdf]

Interleukin-35 suppresses interleukin-9-secreting $CD4^+$ T cell activity in patients with hepatitis B-related hepatocellular carcinoma

Running title: IL-35 suppresses Th9 in HCC

Qian Zhang, Lanlan Yang, Siqi Liu, Mengyao Zhang, Zhenjing Jin *

Digestive Diseases Center, Department of Hepatopancreatobiliary Medicine, The Second Hospital, Jilin University, Changchun, China

* Corresponding author: Zhenjing Jin

E-mail: jljinyu0429@126.com

Supplementary materials and methods

Cellular proliferation assay

10^5 of peripheral blood mononuclear cells (PBMC) from eight patients with hepatitis B-related hepatocellular carcinoma (HCC) were stimulated with recombinant HBV surface antigen (HBsAg, AbD Serotec, Oxford, United Kingdom; 10 μg/mL) in the presence or absence of recombinant human interleukin (IL)-35 (Peprotech, Rocky Hill, NJ, USA; 1 ng/mL) for 24 h. Cellular proliferation was determined by Cell Counting Kit-8 (CCK-8, Beyotime Biotech, Wuhan, Hubei Province, China) according to instructions from the manufacturer.

Flow cytometry

Stimulated PBMCs were transferred to FACS tubes, and were stained with anti-phosphorylated signal transducer and activator of transcription 1 (STAT1) (phosphor S727)-APC (p-STAT1 S727-C6-APC) (Abcam, Cambridge, MA, USA) and p-STAT3 (phosphor Y705)-FITC (p-STAT3 Y705-B12-FITC) (Abcam) for 30 min at room temperature after fixation and permeabilization. Isotype controls for APC and FTIC were used to enable correct compensation and confirm antibody specificity. Acquisitions were performed using Cell Quest Pro Software (BD Bioscience Immunocytometry Systems, San Jose, CA, USA) in a FACS Calibur analyzer (BD Bioscience Immunocytometry Systems). Data were analyzed using FlowJo Software Version 10.0 for Windows (Tree Star, Ashland, OR, USA).

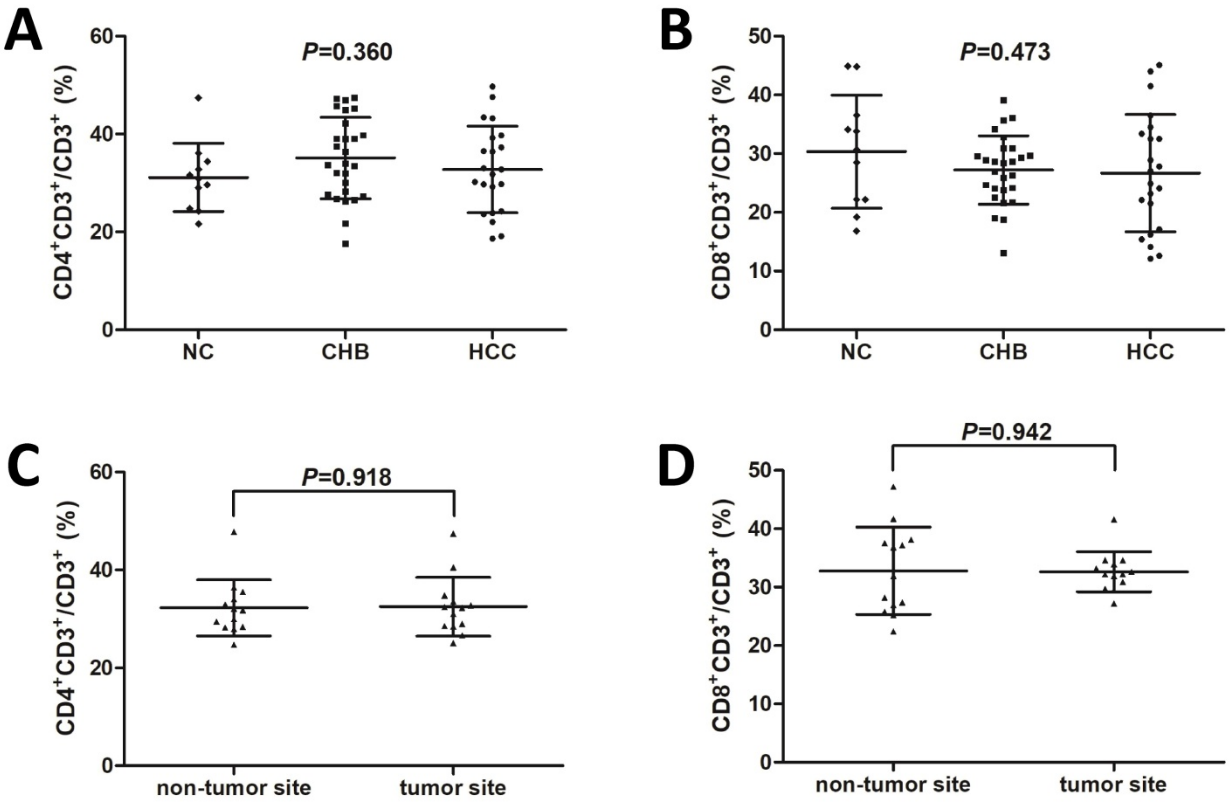

FIGURE S1. The percentage of $CD4^+$ and $CD8^+$ T cells within $CD3^+$ T cells in peripheral bloods and liver residence. Peripheral blood mononuclear cells (PBMCs) were isolated from all enrolled subjects, including normal controls (NC, n=11), chronic hepatitis B (CHB) patients (*n*=27), and hepatitis B-related HCC patients (*n*=22). Intrahepatic lymphocytes (IHLs) were isolated from fresh HCC specimens and non-tumor site liver specimens in thirteen HCC patients (seven in stage A and six in stage B) who underwent hepatic carcinectomy. PBMCs and IHLs were stained with anti-CD3, anti-CD4, and anti-CD8. The percentage of **(A)** $CD4^+CD3^+$ and **(B)** $CD8^+CD3^+$ cells within $CD3^+$ T cells in PBMCs was compared among NC, CHB, and hepatitis B-related HCC patients. The percentage of **(C)** $CD4^+CD3^+$ and **(D)** $CD8^+CD3^+$ cells within $CD3^+$ T cells in IHLs was compared between non-tumor site and tumor site. Significance was assessed using one-way ANOVA or Student *t* test.

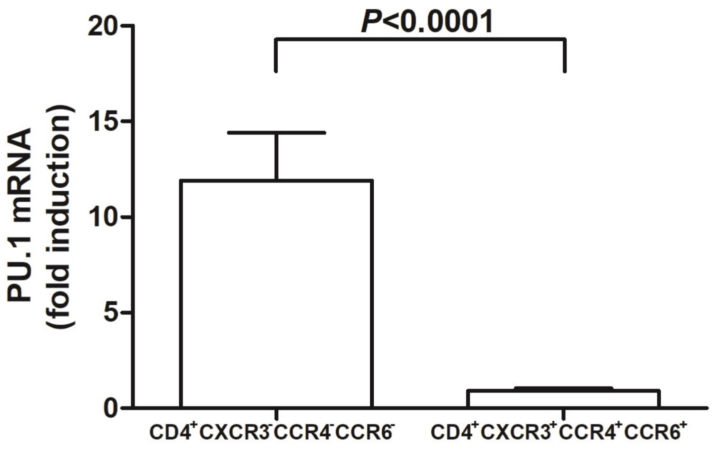

FIGURE S2. PU.1 mRNA expression in CD4$^+$CXCR3$^-$CCR4$^-$CCR6$^-$ cells and CD4$^+$CXCR3$^+$CCR4$^+$CCR6$^+$ cells. Peripheral CD4$^+$CXCR3$^-$CCR4$^-$CCR6$^-$ cells and CD4$^+$CXCR3$^+$CCR4$^+$CCR6$^+$ cells were purified from PBMCs of four normal controls and chronic hepatitis B patients using flow cytometry. PU.1 mRNA expression was quantified using real-time PCR, and was compared between CD4$^+$CXCR3$^-$CCR4$^-$CCR6$^-$ cells and CD4$^+$CXCR3$^+$CCR4$^+$CCR6$^+$ cells. Significance was assessed using Student *t* test.

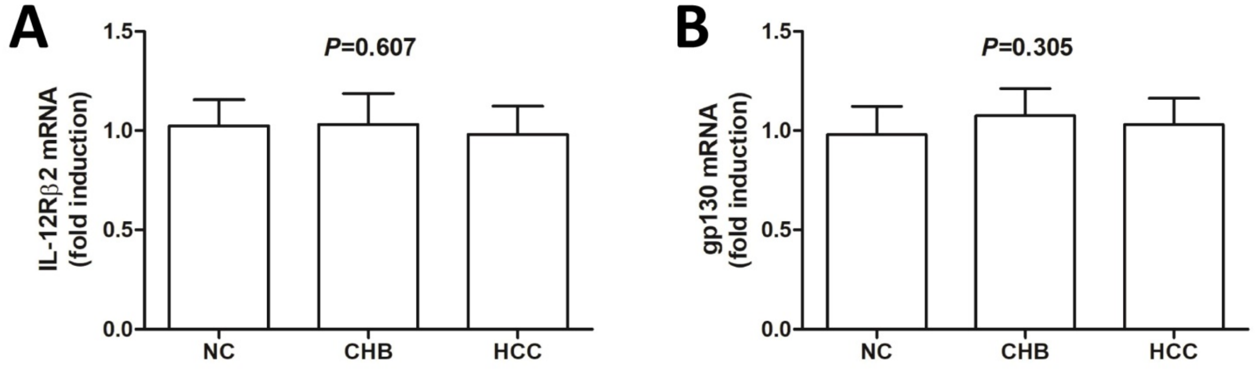

FIGURE S3. IL-35 receptor subunits, IL-12Rβ2 and gp130, mRNA expression in $CD4^{+}CXCR3^{-}CCR4^{-}CCR6^{-}$ cells. Peripheral $CD4^{+}CXCR3^{-}CCR4^{-}CCR6^{-}$ cells were from PBMCs nine normal controls (NC), fifteen chronic hepatitis B (CHB) patients, and fifteen hepatitis B-related HCC patients using flow cytometry. **(A)** IL-12Rβ2 and **(B)** gp130 mRNA expression was quantified using real-time PCR, and was compared among three groups. Significance was assessed using one-way ANOVA.

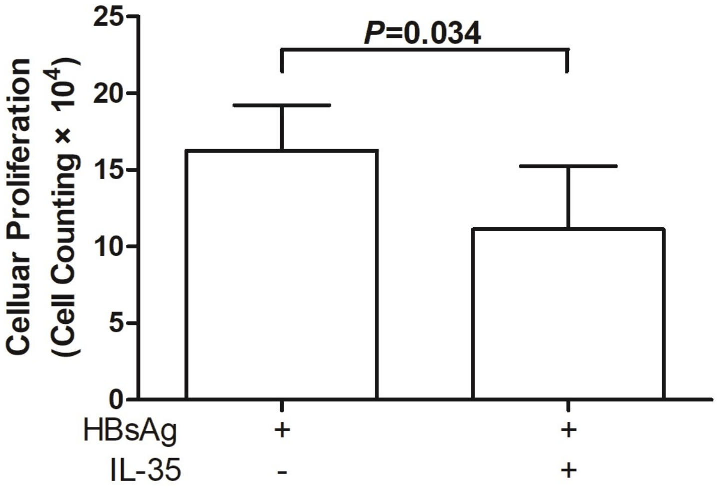

FIGURE S4. Cellular proliferation in response to IL-35 stimulation. 10^5 of peripheral blood mononuclear cells (PBMC) from eight patients with hepatitis B-related hepatocellular carcinoma (HCC) were stimulated with recombinant HBV surface antigen (HBsAg, 10 μg/mL) in the presence or absence of recombinant human interleukin (IL)-35 (1 ng/mL) for 24 h. Cellular proliferation was determined by Cell Counting Kit-8 (CCK-8), and was compared between two groups. Significance was assessed using Student *t* test.

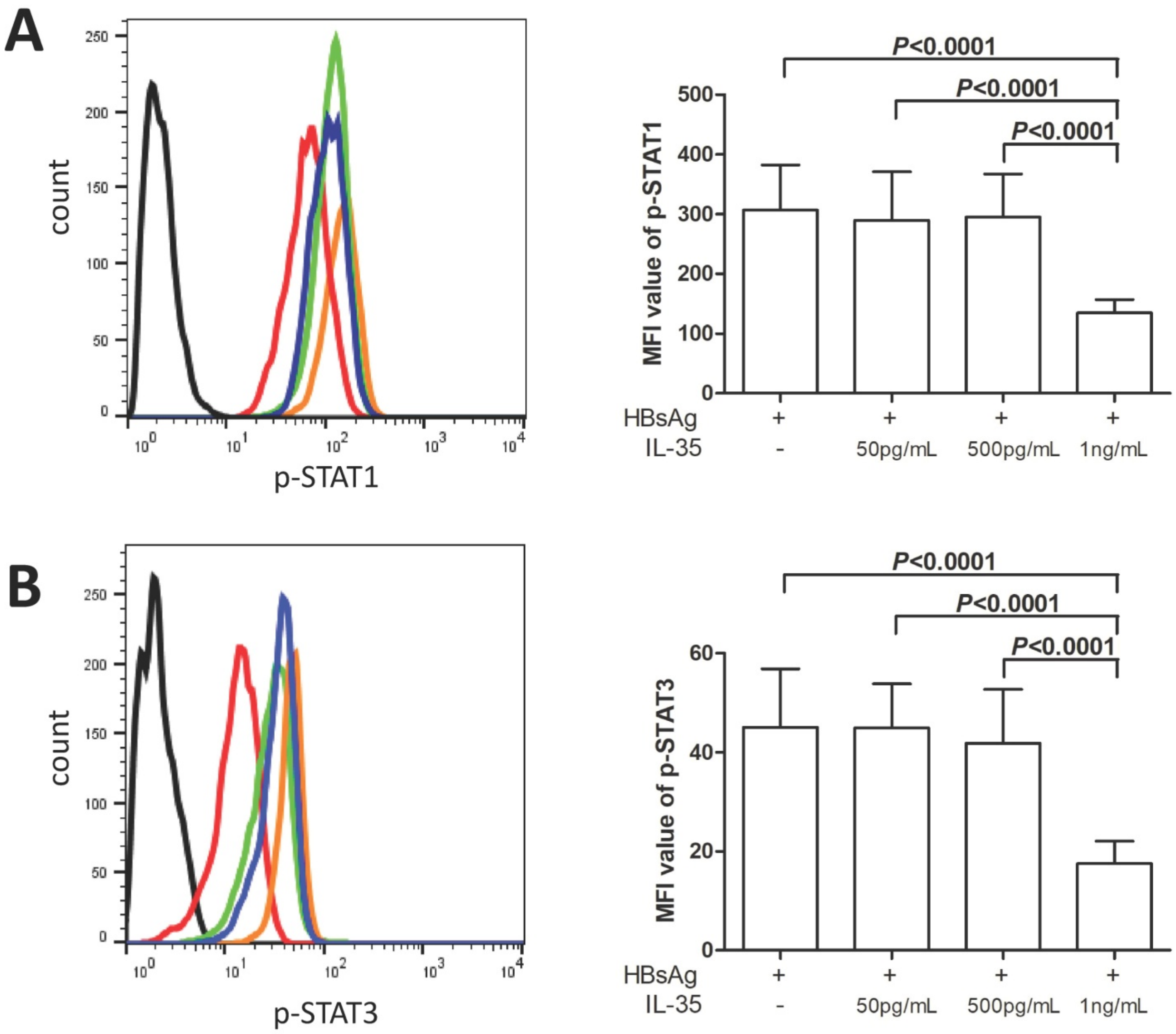

FIGURE S5. Signal transducer and activator of transcription 1 (STAT1) and STAT3 phosphorylation in response to IL-35 stimulation. 10^5 of peripheral blood mononuclear cells (PBMC) from seven chronic hepatitis B (CHB) patients and eight patients with hepatitis B-related hepatocellular carcinoma (HCC) were stimulated with recombinant HBV surface antigen (HBsAg, 10 µg/mL) in the presence or absence of recombinant human interleukin (IL)-35 (50 pg/mL, 500 pg/mL, or 1 ng/mL) for 24 h. Cells were stained with anti-p-STAT1 S727-C6-APC and anti-p-STAT3 Y705-B12-FITC, and were analyzed by flow cytometry. The representative histograms for **(A)** p-STAT1 and **(B)** p-STAT3 were shown. The black line represented the isotype controls, the orange line represented cells without IL-35 stimulation, the blue line represented cells with 50pg/mL of IL-35 stimulation, the green line represented cells with 500pg/mL of IL-35 stimulation, and the red line represented

cells with 1ng/mL of IL-35 stimulation. The mean fluorescence intensity (MFI) corresponding to **(A)** p-STAT1 and **(B)** p-STAT3 was compared between groups. Significance was assessed using one-way ANOVA and SNK-*q* test.
